# Vitamin D Deficiency and Exocrine Pancreatic Insufficiency: An Analysis Carried Out in Orthogeriatric Patients (VIDEP.org)

**DOI:** 10.3390/jcm14155558

**Published:** 2025-08-07

**Authors:** Pavol Mikula, Matthias Unseld, Hans Jürgen Heppner

**Affiliations:** 1 Clinic for Geriatrics and Geriatric Day Clinic, Bayreuth Hospital, Medical Campus Upper Franconia (MCO), Preuschwitzer Strasse 101, 95448 Bayreuth, Germany; hans.heppner@klinikum-bayreuth.de; 2 Chair of Geriatrics, Friedrich-Alexander University Erlangen-Nuremberg, Hugenottenplatz 6, 91054 Erlangen, Germany; 3 Department of Clinical Research SBG, Academy for Ageing Research, Haus der Barmherzigkeit, 1160 Vienna, Austria; matthias.unseld@hb.at; 4 Division of Palliative Medicine, Department of Medicine I, Medical University of Vienna, 1090 Vienna, Austria

**Keywords:** vitamin D, orthogeriatric patient, exocrine pancreatic insufficiency

## Abstract

**Introduction:** Vitamin D deficiency, a reversible cause of osteoporosis, is increasingly prevalent, showing varying degrees of severity that are notably pronounced among the growing population of multimorbid elderly patients. Given that the aging pancreas undergoes senescent processes leading to impaired function—which negatively impacts enteral vitamin D absorption and, consequently, elderly bone metabolism—a specific diagnostic and treatment approach is crucial. Our study aimed to determine the prevalence of vitamin D deficiency and exocrine pancreatic insufficiency (EPI) in orthogeriatric patients. We also evaluated differences in vitamin D deficiency severity between patients with normal and impaired pancreatic function. Furthermore, a short-term monitoring of vitamin D level increases after 12 days of substitution therapy in both groups aimed to inform osteoanabolic therapy for specific high-fracture-risk patients, assessing the influence of pancreatic function on substitution efficacy. **Methods:** We conducted a retrospective, monocentric cohort study, evaluating data from all patients hospitalized with manifest osteoporosis in an orthogeriatric department during a six-month spring/summer period. Demographic data, relevant comorbidities, the type of fracture, the amount of faecal elastase 1 (CALEX^®^ Cap Bühlmann), and the serum levels of 25-hydroxyvitamin D (25(OH)D) were assessed. **Results:** We found a high prevalence (70.6%) of vitamin D deficiency (25(OH)D < 30 µg/L) among all orthogeriatric patients. Of these, 16% met the criteria for mild to severe EPI. The group with normal exocrine pancreatic function showed a higher average vitamin D value, and their increase in vitamin D levels following short-term substitution was up to 100% greater compared to the group with impaired pancreatic function. Notably, 69% of women and 20% of men met the therapeutic threshold for specific osteoanabolic osteoporosis therapy, even without a T-score. **Conclusions:** Our findings reveal a very high prevalence of vitamin D deficiency and a high prevalence of EPI in orthogeriatric patients. Those with impaired exocrine pancreatic function exhibit lower baseline vitamin D levels and a diminished capacity for vitamin D absorption during short-term monitoring. These results have significant clinical implications for osteoporotic therapy, given that a substantial proportion of patients, particularly women, meet the criteria for specific osteoanabolic treatment.

## 1. Introduction

Multiple factors are causing the increasing prevalence of vitamin D deficiency in today’s population. A significant factor is the mismatch between the human need for sunlight exposure for cutaneous exposure to ultraviolet B (UVB) radiation and the typical patterns of modern life. In addition, the use of photoprotective measures, such as the application of sunscreen, contributes to a reduction in UVB-induced vitamin D synthesis. These measures are highly indicated in order to protect against skin cancer, but they limit physiological vitamin D production. Resulting hypovitaminosis D (vitamin D deficiency) can lead to a large number of health consequences, as vitamin D has a pleiotropic effect on numerous physiological processes, including the regulation of proper calcium and phosphate levels in the body. As a standard marker, 25-hydroxyvitamin D (25(OH)D), also known as calcidiol, is measured in the serum; this is the precursor of the active form of vitamin D (calcitriol). A vitamin D deficiency is assumed if the 25(OH)D concentration in serum or plasma is below 30 µg/L (30 ng/mL or 75 nmol/L) [1,2]. Accordingly, the suboptimal range (insufficiency) is between 20 and 29 µg/L, while 12 to 19 µg/L indicates a deficiency, and a severe deficiency corresponds to values below 12 µg/L. Potentially harmful hypervitaminosis is defined as values between 60 and 149 µg/L, and toxic values with a risk of hypercalcaemia are to be expected from 150 µg/L. The worldwide prevalence of vitamin D deficiency varies. Prevalence rates for severe vitamin D deficiency, defined as 25(OH)D < 12 µg/L, of 5.9% are reported in the USA [3], 7.4% in Canada [4], and 13% in Europe [5]. Specific patient cohorts have a significantly increased prevalence of hypovitaminosis D; patients with chronic renal insufficiency and patients with liver or pancreas disorders in particular display a remarkably high prevalence of vitamin D deficiency ranging from 85 to 99% [6,7,8].

Vitamin D

Together with vitamins A, E, and K, vitamin D belongs to the group of fat-soluble vitamins. In view of the fact that the receptors for it are widespread in the human body and present in almost all tissues and organs, this indicates that vitamin D performs a wide range of functions in addition to its role in calcium metabolism. The ability to obtain vitamin D from both sunlight and food is an evolutionary advantage that ensures adaptability to wide-ranging environmental conditions. Around 80–90% of the required vitamin D is produced as a result of skin exposure to UVB radiation. The remaining 10–20% of vitamin D comes from dietary sources [9].

Endogenous synthesis in the skin with the aid of sunlight

From a chemical point of view, vitamin D is a group of fat-soluble vitamins. This prohormone first needs to be converted in the human body in multiple steps into its active form—calcitriol. The provitamin 7-dehydrocholesterol formed in the human body is converted with the aid of UVB radiation to calciol. The thermodynamically unstable previtamin D3 is formed, and this is then converted to the actual vitamin D3 (cholecalciferol or calciol). Around 20% of the starting amount of the previtamin D3 is converted in this way. The calciol (vitamin D3) then passes into the blood and is bound to vitamin D-binding protein (DBP) and transported to the liver for hydroxylation, where the 25-hydroxyvitamin D (25(OH)D)—calcidiol—is produced as the precursor of the active form of vitamin D. Calcidiol as the storage form of vitamin D3 has a half-life of around 19 days.

With increasing age, the ability of the skin to produce vitamin D also deteriorates. From a pathophysiological perspective, this is caused by the reduced concentration of 7-dehydrocholesterol. In some studies, a synthesis reduction of up to 50% was documented [10]. Other studies estimate that the ability of the skin to form vitamin D is reduced by a factor of 3 in comparison to that of a 20-year-old person [11]. In addition, older people also spend less time outdoors, which further reduces exposure to sunlight and therefore vitamin D synthesis.

Enteral absorption of vitamin D

Vitamin D can also be obtained from dietary sources. However, there are only a few natural dietary sources that contain enough vitamin D_2_ and D_3_. For example, vitamin D_3_ (cholecalciferol) is contained in oily fish such as salmon or herring. Cod liver oil, egg yolk, or beef liver are further sources, albeit with highly variable concentrations. Vitamin D_2_ (ergocalciferol) is contained, for example, in mushrooms. Enteral absorption of vitamin D is a complex process in which pancreatic lipase plays a crucial role in the absorption of vitamin D. As a result of the formation of micelles and induced by pancreatic lipase, vitamin D can be absorbed via the brush border membrane of the enterocytes. Within the enterocytes, vitamin D is incorporated into chylomicrons. These are then released from the enterocytes into the lymphatic vessels. Within the bloodstream, vitamin D is bound to vitamin D-binding protein (DBP) and, once absorbed in this way, the vitamin D then present in the bloodstream is twice hydroxylated until the active form is present, i.e., 1,25-dihydroxycholecalciferol (calcitriol).

Pancreas and exocrine pancreatic insufficiency

The pancreas plays a crucial role in lipid digestion by synthesizing and secreting pancreatic lipase, which therefore makes it indispensable in the absorption of fat-soluble vitamins [12]. The formation of micelles already described above is a critical step in intestinal fat absorption [12]. Links have been established between exocrine pancreatic insufficiency and vitamin D [13,14,15]. The first documented indirect indications of potential exocrine pancreatic insufficiency with clinically dominant and measurable steatorrhoea and osteoporosis as a consequence are dated to the year 1936, with further case reports in the subsequent years [16,17]. The enzyme elastase 1 was not discovered until 1949 and was first isolated in 1952 [18].

The measurement of faecal pancreatic elastase 1 is a very reliable method for assessing exocrine pancreatic function. Due to their non-invasive nature and high accuracy, these tests established themselves as a valuable instrument for assessing pancreatic function and for diagnosing diseases such as exocrine pancreatic insufficiency [19]. Just like all other organs, the pancreas is subject to an ageing process. Today, these changes are well described both with the aid of imaging using abdominal ultrasound, endoscopic ultrasound, or CT/MRT scans and with the aid of microscopic pathology. Faecal elastase 1 concentrations correlate negatively with age, with a prevalence of up to 20% among healthy older patients without any pre-existing gastroenterological diseases [20,21]. Table 1 shows standard values for the determination of faecal elastase 1.

Faecal concentrations of elastase 1 above 200 µg per g of stool are regarded as normal. Faecal concentrations of elastase 1 between 100 and 200 µg per g of stool indicate mild to moderate exocrine pancreatic insufficiency, and severe exocrine pancreatic insufficiency is present at concentrations below 100 µg per g of stool.

Aim and objectives

The VIDEP.org-study aims to (i) determine the prevalence of vitamin D deficiency in orthogeriatric patients independently of whether or not substitution had already taken place before admission to hospital; (ii) investigate the prevalence of exocrine pancreatic insufficiency in this patient group and evaluate whether the vitamin D deficiency is more pronounced when exocrine pancreatic insufficiency is present; and (iii) record any changes in vitamin D levels after 12 days of substitution therapy.

## 2. Materials and Methods

All patients consecutively hospitalized in the orthogeriatric department were included in the retrospective, monocentric study, and based on the medical records, the data were evaluated throughout the spring/summer period of 6 months. The time period was chosen outside of the winter months to rule out seasonal hypovitaminosis. Where indicated, substitution during the retrospective observation period was administered with a combined preparation of 1000 mg calcium with 800 IU vitamin D3 (Calcium Sandoz D Osteo; Hexal AG, Holzkirchen, Germany) taken once per day orally.

In addition to demographic data, the relevant comorbidities, the type of fracture, the amount of faecal elastase 1 (CALEX® Cap, Bühlmann Laboratories AG, Schönenbuch, Switzerland) and the level of 25-hydroxyvitamin D (25(OH)D) in the blood were assessed at the start of the orthogeriatric treatment—but before the start of vitamin D substitution, where indicated, during the inpatient treatment. Any outpatient vitamin D substitution that had already begun was continued. The quantitative determination of elastase 1 was possible up to 500 µg/g in the laboratory, which also permitted evaluation in the higher standard range. Stool samples that were contaminated with urine and stool samples obtained after administration of laxatives were excluded in order to minimise the likelihood of false-positive results. Parathormone level in serum on the day of determination of the 25-hydroxyvitamin D (25(OH)D) level, the estimated glomerular filtration rate (eGFR) using the CKD-EPI (Chronic Kidney Disease Epidemiology) equation, the total calcium, serum albumin and calcium corrected for albumin were determined; the ionised calcium was measured via venous blood gas analysis either as part of pre-surgical preparations or after transfer to the orthogeriatric department. In line with the current osteoporosis guidelines of Dachverband Osteologie e.V. [22], the relevant secondary diagnoses—which were used to determine the fracture risk constellation and the risk gradient for determination of the therapy indication—were recorded and evaluated. On day 12 of the in-patient stay, the 25-hydroxyvitamin D (25(OH)D) level was checked, and the average sex-specific T-score was determined in a small subgroup. We have not utilized any artificial intelligence (AI) tools, such as large language models, image generators, or data synthesis platforms, to generate any portion of the content.

Statistics and ethics

IBM SPSS Statistics (Version 29.0.1.0; IBM Corp., Armonk, NY, USA) and Microsoft Excel LTSC MSO (Version 16.0.14334.20136 64-Bit; Microsoft Corp., Redmond, WA, USA) were used for the calculations. The data was collected, cleaned up, and transferred. The quantitative data analysis was performed with the aid of descriptive statistics, whereby the measures of central tendency, measures of dispersion, and frequencies were calculated. Afterwards, an inferential statistical analysis was carried out using a Welch test.

We hereby declare that all text, data, and graphics submitted as part of this article are our own original work. We have not utilized any artificial intelligence (AI) tools, such as large language models, image generators, or data synthesis platforms, to generate any portion of the content. All sources of information have been appropriately cited and referenced.

## 3. Results

During the observation period, a total of 193 orthogeriatric inpatients with typical fractures for osteoporosis were included. The average age was 82.9 year old (maximum 103, minimum 64, median 84, modal value 85). A total of 28% (55 of 193) of the patients were male, and 72% (138) were female. In 170 patients it was possible to measure the 25-hydroxyvitamin D (25(OH)D) level. Four patients died during the observation period and nineteen required temporary intensive care treatment and were not included in the evaluation. In 124 patients it was possible to determine the elastase 1 levels in stool.

Vitamin D

In 170 patients it was possible to evaluate 25-hydroxyvitamin D (25(OH)D) in accordance with the defined criteria. The average serum value was 21.9 µg/L, the median value 19 µg/L. In three patients (1.7%) the 25-hydroxyvitamin D (25(OH)D) values were in the potentially harmful range (60.0–149 µg/L). Only 47 patients (27.6%) were in the target range (30.0–59.0 µg/L), 31 (18.2%) displayed insufficiency (20.0–29.0 µg/L), 33 (19.4%) displayed a verified vitamin D deficiency (12.0–19.0 µg/L) and 56 (33%) displayed a severe deficiency (<12 µg/L). As a result, 70.6% (120 out of 170) of the patients displayed varying levels of insufficiency or deficiency in relation to the target range for the 25-hydroxyvitamin D (25(OH)D) level of <30 µg/L.

Elastase 1 in stool

Elastase 1 in stool as an indicator of exocrine pancreatic function was determined in 124 patients in accordance with defined criteria and exclusion criteria. A severe deficiency with concentrations below 100 µg/g was displayed by 10 patients (8%). Mild to moderate exocrine pancreatic insufficiency was also present in 10 patients (8%). A total of 16% of the patients, therefore, met the criteria for mild to severe exocrine pancreatic insufficiency. The other 104 cases investigated (84%) displayed values above 200 µg/g and were therefore in the normal range.

Analysis of vitamin D levels in connection with exocrine pancreatic function

(A) 25-hydroxyvitamin D (25(OH)D) levels in the group of patients with elastase 1 concentrations > 200 µg/g (n = 88).

The average vitamin D level for elastase 1 values > 200 µg/g was 23.4 µg/L and was 7% higher than the overall average (21.9 µg/L). Among the patients in this group, 3 (3%) displayed hypervitaminosis D, 26 (30%) were in the target range, and 59 (67%) of the patients were below the normal range with varying degrees. Table 2 shows a summary of the values.

(B) 25-hydroxyvitamin D (25(OH)D) levels in the group of patients with elastase 1 concentrations < 200 µg/g (n = 15).

**Figure 1:**
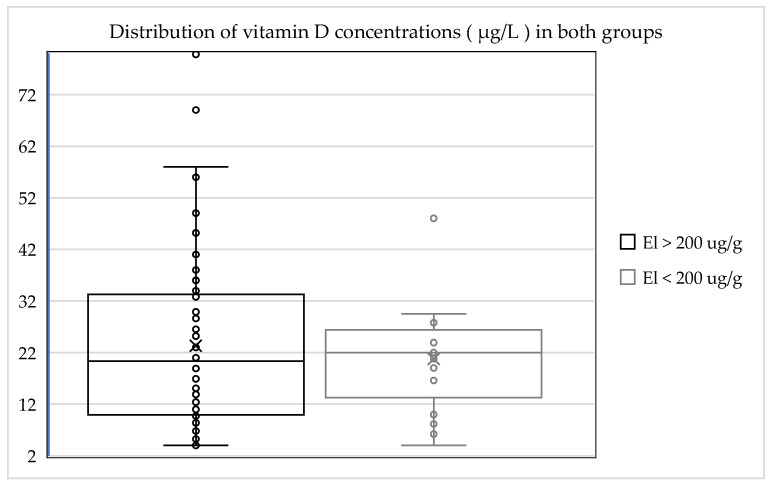
Distribution of vitamin D concentrations in the different groups.

The average 25-hydroxyvitamin D (25(OH)D) level was 20.75 µg/L and was therefore 11% lower than in the group with normal exocrine pancreatic function (23.4 µg/L). There was no hypervitaminosis in this group. In this group only one patient (6%) was in the target range, which was 27% (25) less than in comparison to the investigated cohort with normal exocrine pancreatic function. A total of 94% (14 of 15) of those investigated were below the target range for vitamin D. A summary of this is shown in Table 3, and the distribution of vitamin D concentrations in both groups is presented in Figure 1. 

Both patient groups displayed insufficiency in relation to the target range for 25-hydroxyvitamin D (25(OH)D) levels. The group of patients with normal exocrine pancreatic function displayed a higher average value, and the insufficiency in terms of 25-hydroxyvitamin D (25(OH)D) levels in the group with exocrine pancreatic insufficiency was more pronounced by 11%. A total of 94% (14 of 15) of the patients with exocrine pancreatic insufficiency were below the target range for vitamin D. Only 6% (1 of 15) were within the normal range. Hypervitaminosis was not observed in any cases in this group.

**Figure 2:**
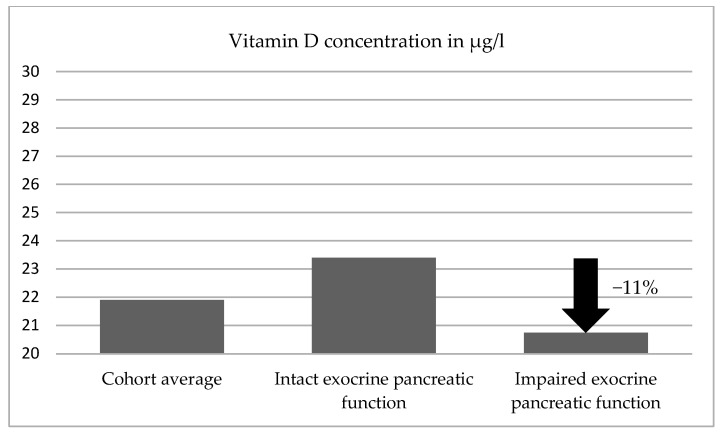
Degree of vitamin D deficiency and comparison between the different groups.

Inferential statistics revealed that the mean vitamin D concentration in group 1 (normal elastase 1) was 23.4 ± 17.13 μg/L (mean ± SD), while in group 2 (low elastase 1), it was 20.7 ± 11.35 μg/L. The calculated *p*-value for this difference was 0.491. The different groups are compared in Figure 2.

Vitamin D increase after 12 days of substitution therapy

On day 12 after the start of oral substitution therapy, the 25-hydroxyvitamin D (25(OH)D) level was measured. Due to unforeseen events, varying disease courses, and false positive samples, we were able to acquire the necessary data from 38 patients without exocrine pancreatic insufficiency and 10 patients with confirmed exocrine pancreatic insufficiency. Its increase in concentration was then compared between the groups with and without exocrine pancreatic insufficiency, relative to their initial vitamin D levels. In the group of patients with normal concentrations of faecal elastase 1, there was a rise in the 25-hydroxyvitamin D (25(OH)D) level by on average 7.2 µg/L, which corresponded on average to an increase of the starting value by 120%. In the group of patients with exocrine pancreatic insufficiency, there was a rise in the 25-hydroxyvitamin D (25(OH)D) level by on average 3.6 µg/L, which corresponded on average to an increase of the starting value by 23%. Between the different groups, with regard to exocrine pancreatic function and the correlating vitamin D level in serum, there was a 100% difference in relation to the increase of the 25-hydroxyvitamin D (25(OH)D) level under substitution therapy during the observation phase. With inferential statistics, we found a significant difference in the average vitamin D increase under substitution between the two groups (t(15.77) = −2.48, *p* = 0.024). Baseline characteristics and vitamin D increase are shown in Table 4.

Analysis of kidney function

The average eGFR was 60.8 mL/min (median: 60 mL/min); two patients had a chronic need for dialysis and were, due to their specific nephrologic treatment regarding calcium, vitamin D, and parathormone target levels, not included in the calculations. A total of 12% (17 of 145) had an eGFR ≥ 90 mL/min, 42.5% (62 of 145) had an eGFR between 60 and 89 mL/min, and 34.5% (50 of 145) of the patients had an eGFR in the range between 30 and 59 mL/min. In terms of their kidney function, 11% of the patients (16 of 145) were in stage G4 of chronic renal insufficiency based on KDIGO 2024 (15–29 mL/min) as shown in Table 5.

The subgroup investigation regarding the different therapy indications (antiresorptive vs. osteoanabolic osteoporosis therapy) showed a hip-area T-score determined for the risk constellation calculation of on average −1.5 and a median value of −1.3; here, a clear negative correlation with rising age could be observed. Either on account of their age or in combination with relevant secondary diagnoses, 69% of all women (96 of 138) had reached the therapeutic threshold for specific osteoanabolic osteoporosis therapy even without the T-score. For 27 of these (28%), this was solely on account of their age. This applied to 19.5% (27 of 138) of all women. Of the 55 men, 11 (20%) were above the 10% therapeutic threshold on account of their age and the relevant specific secondary diagnoses. The small subgroup with an available T-score primarily included women (8 of 9). Six of nine female patients (69%) have reached the therapeutic threshold for specific osteoanabolic osteoporosis therapy based on consideration of age, patient-specific relevant secondary diagnoses, and the patient-specific T-score.

## 4. Discussion

Orthogeriatric patients represent a specific and particularly vulnerable patient group due to their multimorbidity, polymedication, and typical geriatric syndromes. Physiological aging of organs in these patients leads to reduced resilience, diminished regeneration capability, and functional decline. This physiological aging also affects the gastrointestinal tract (GIT), resulting in reduced calcium absorption, decreased intestinal motility, and impaired nutrient absorption. Furthermore, both endocrine and exocrine pancreatic secretions decrease. This presents a risk not only for malnutrition but also for vitamin D malabsorption. With the increasing aging of the population, osteoporotic fractures are also a common age-related disease. The resulting need for treatment is associated with significantly higher morbidity and mortality and also represents a high economic burden on the healthcare system [23].

In older age, osteoporosis is frequently, but not exclusively, attributed pathophysiologically to vitamin D deficiency. Several studies have impressively highlighted the importance of vitamin D in the prevention of various diseases. In the area of cancer diseases, the link between vitamin D levels and colorectal cancer in particular has been intensively researched. Here, a clear protective effect was shown: the higher the 25-hydroxyvitamin D (25(OH)D) level, the lower the risk of disease. With a concentration in serum of ≥33 µg/L, the incidence of colorectal cancer was halved in comparison to ≤12 µg/L [24]. With breast cancer the results are less clear-cut, and no link was established for prostate cancer [25,26]. In orthogeriatric patients, vitamin D deficiency is a chronic condition with varying symptoms and is regarded as a contributing cause of osteoporosis. A link between high levels of 25(OH)D in serum and increased bone mineral density has been demonstrated in studies [27,28]. Furthermore, it has been shown that daily supplements of vitamin D can reduce the risk of hip fractures by around 25% [27]. Analysis of 675 bone biopsies showed pathological changes to the bone mineralisation only in patients with 25(OH)D values in serum of below 30 µg/L [28]. The authors concluded from this that a 25(OH)D level in serum of at least 30 µg/L is necessary for optimum bone health [28]. Based on the findings of meta-analyses, which support a clear link between low vitamin D levels and an increased fracture risk in older patients, ensuring an adequate supply of vitamin D forms an integral part of the basic measures used in osteoporosis therapy [29]. However, this is subject to adequate absorption. Here, the investigations have shown that the exocrine pancreatic function of the patients during the observation period plays a crucial role.

The prevalence of vitamin D deficiency varies between different patient groups, which is why the percentage stated in the specialist literature can fluctuate between 20 and 90% [6,7,8,30]. This is also reflected in the data collected from the investigated patient groups and, as a consequence, this entails a structured supplementation. However, there is no uniform therapy recommendation for the treatment of vitamin D deficiency; this applies to both the dosage and the therapy regimen, as well as to the issue of how quickly a deficit should be corrected, e.g., after a fracture has occurred or in cases of imminent osteoporotic fracture. In addition, this retrospective analysis looks at the extent to which intestinal malabsorption and excretion disorders are significant. It is known that after a first osteoporotic fracture, the risk of further fractures is significantly increased, particularly in the first two years [31]. Even without previous fractures, the risk of fracture can be calculated today with the aid of various scores [32], and it has been predicted that the number of people with a high fracture risk will double worldwide between 2010 and 2040 [33].

As with almost all diseases, individual target setting based on the extent of the deficit and the comorbidities will be necessary here, and in terms of pathophysiology, there are different targeting strategies. On the one hand, this includes a reduction of parathormone by the maximum possible amount in order to eliminate long-term negative consequences of hyperparathyroidism. In order to achieve the optimum endocrinological effect here, a 25-hydroxyvitamin D (25(OH)D) level of at least 30 µg/L appears to be necessary [34]. On the other hand, adequate calcium absorption comes to the fore. It is to be assumed that a pronounced vitamin D deficiency with concentrations < 4.4 µg/L represents insufficient substrate for further conversion to 1,25-dihydroxyvitamin D, regardless of the parathormone level [35]. With an extremely low value of vitamin D, the mechanism to compensate for this is an increased production of parathormone (secondary hyperparathyroidism).

Our investigated patients reflected geriatric multimorbidity and had experienced an osteoporotic fracture. The prevalence of 25-hydroxyvitamin D (25(OH)D) deficiency was 70.6%, combined with a prevalence of exocrine pancreatic insufficiency of 16%. While a vitamin D deficiency was factually detectable in both groups, and was quantitatively 11% more pronounced in the group with exocrine pancreatic insufficiency, we did not find a statistically significant difference in baseline vitamin D levels between the groups. Specifically, the mean vitamin D level in the group with normal elastase 1 was 23.4 µg/L (SD 17.13), and in the group with low elastase 1, it was 20.7 µg/L (SD 11.35), with a *p*-value of 0.491. Despite this lack of statistical significance between groups, the actual presence of vitamin D deficiency in both groups and the observable quantitative difference is of significant clinical relevance for therapy planning. This observation, coupled with the understanding that an intact gastrointestinal tract and intact hepatic, renal, and metabolic functions are fundamental for producing active vitamin D in sufficient quantities under adequate sun exposure and enteral nutrition, highlights the importance of addressing this deficit. It is, however, crucial to acknowledge that the observation period was short and thus limited, preventing reliable conclusions about medium or long-term results. Furthermore, 25(OH)D level measurements are known to be subject to fluctuations of up to 30% [36].

### 4.1. Clinical Relevance of Hormone Levels and Calcium Homeostasis

Our analysis of patient data revealed several critical points regarding patient characteristics and therapeutic responses. We found a statistically significant lower albumin level in the group with abnormal exocrine pancreatic function (*p* = 0.003), supporting the hypothesis that exocrine pancreatic dysfunction may lead to malabsorption. Although total calcium levels did not show a statistically significant difference between groups (*p* = 0.799), albumin-corrected calcium levels were statistically significantly lower in the group with exocrine pancreatic insufficiency (*p* = 0.012). This indicates that once the influence of serum albumin on calcium measurement is accounted for, a genuine difference in “physiologically relevant” calcium status emerges, which was obscured by uncorrected total calcium readings. Despite these significant differences in albumin and albumin-corrected calcium, ionized calcium (*p* = 0.348) showed no statistically significant difference between the groups. This suggests that the body of the patients, even with exocrine pancreatic insufficiency, was apparently able to maintain physiologically active calcium levels (ionized calcium) relatively stable and similar to the control group.

Additionally, we found that both patient groups had elevated mean parathormone levels (65.7 ± 35.7 in exocrine pancreatic insufficiency and 69.4 ± 33.8 in normal pancreatic function). These values are notably above the typical upper reference ranges for parathormone, indicating a high prevalence of (secondary) hyperparathyroidism in this orthogeriatric cohort. This is a crucial finding for post-hospital care, especially considering the imminent fractures, as hyperparathyroidism is a contraindication for many osteoanabolic therapies that these patients might require in the future. It is worth noting that we found no statistically significant difference in the manifestation of this hyperparathyroidism between the two groups (*p* = 0.777), which aligns with the non-significant differences in ionized calcium, suggesting that the body did not require further significant PTH adjustment between the groups to regulate acute calcium homeostasis.

### 4.2. Response to Vitamin D Supplementation

After the initiation of oral supplementation with 800 IU 25(OH)D and 1000 mg calcium, laboratory control tests after 12 days showed a statistically significant increase in 25(OH)D levels in the group without exocrine pancreatic insufficiency (mean 7.2 ± 4.7 µg/L) compared to the group with exocrine pancreatic insufficiency (mean 3.6 ± 2.3 µg/L) with a *p*-value of 0.024. This statistically significant difference in mean increase between the two elastase 1 groups suggests that the ability to absorb or process vitamin D is potentially influenced by pancreatic function. The considerably higher increase in the group with supposedly better pancreatic function indicates more efficient absorption or metabolism of the supplemented vitamin D. This implies that in patients with exocrine pancreatic insufficiency, standard vitamin D supplementation may be less effective, at least in the short term.

### 4.3. Outlook and Recommendations

The lack of statistical significance in baseline vitamin D levels between the two elastase 1 groups is primarily due to the very small patient sample size (n = 15) in the low elastase group, which also included patients receiving outpatient hormone supplementation with vitamin D under varying therapy regimens. Nevertheless, the analysis reveals a clinically relevant difference in vitamin D levels and especially their increase between the two groups. In this context, follow-up studies with pancreatic hormone supplementation are planned.

Based on these findings, it becomes particularly important for our investigated group of geriatric patients with multimorbidity and imminent fractures to quickly examine exocrine pancreatic function and, in cases of insufficiency, initiate substitution to enable rapid bone mass buildup. Examples from gastroenterological studies with much younger patients suffering from chronic pancreatitis in an advanced stage with demonstrable exocrine pancreatic insufficiency provide answers to what happens if this vitamin D malabsorption remains untreated in the long term. A meta-analysis of 10 studies estimated the pooled prevalence of both osteopenia and osteoporosis to be 65% [15]. Furthermore, patients with chronic pancreatitis (CP) have an increased risk of low-trauma fractures [37].

The recommendations of the German DVO (Dachverband Osteologie) in the osteoporosis guidelines do not primarily consider patients with renal insufficiency with eGFR < 30 mL/min/1.73 m^2^, which corresponds to stages IV and V [22]. However, the determination of renal function does influence the further approach regarding therapy planning. The indication for switching from cholecalciferol (vitamin D_3_) to calcitriol in patients with chronic kidney disease (CKD) is determined primarily by the stage of progression of the CKD and the residual renal 1α-hydroxylase activity. Regular determination of kidney function is therefore essential, as the collected data displays a large range of variation in terms of eGFR values. Most current guidelines for treatment of osteoporosis recommend measuring the vitamin D level before the start of a specific osteoporosis therapy, including osteoanabolic osteoporosis therapy, and correcting any deficiency found. Target values for the vitamin D level are generally at least 30 µg/L. 

## 5. Conclusions

**Figure 3:**
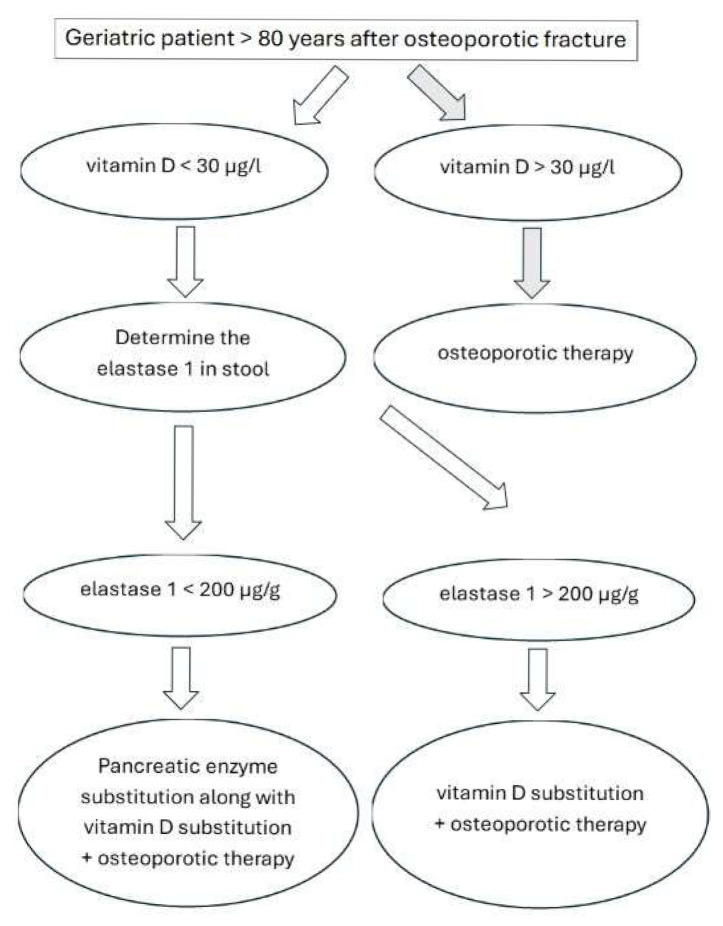
Recommendations for geriatric patients after osteoporotic fracture. Vitamin D = 25-hydroxyvitamin D = 25(OH)D. Osteoporotic therapy as well as pancreatic enzyme substitution according to local guidelines.

The prevalence of vitamin D deficiency in this specific cohort of orthogeriatric patients is remarkably high at 70%, with exocrine pancreatic insufficiency also prevalent at 16%, notably above that found in the general population. Our analysis revealed no statistically significant difference in baseline vitamin D levels between groups, despite a quantitatively 11% more pronounced deficiency in those with exocrine pancreatic insufficiency. However, we found a statistically significantly lower albumin level (*p* = 0.003) and albumin-corrected calcium levels (*p* = 0.012) in the group with abnormal exocrine pancreatic function. Crucially, after supplementation, there was a statistically significant difference in the mean increase of 25(OH)D levels (*p* = 0.024), with a considerably higher increase in the group without exocrine pancreatic insufficiency (mean 7.2 ± 4.7 µg/L vs. 3.6 ± 2.3 µg/L). Both groups presented with elevated parathormone levels, though with no statistically significant difference between them. These findings underscore the importance of routinely measuring vitamin D values, elastase 1, and parathormone in orthogeriatric inpatients to optimize management and improve bone health outcomes, especially given the attenuated response to vitamin D supplementation observed in patients with pancreatic insufficiency. A concise summary offering practical guidance is provided in Figure 3.

## Figures and Tables

**Table 1:** Standard values of faecal elastase 1.

Finding	Faecal Elastase 1 (µg per g of Stool)
Normal	>200 µg/g
Mild to moderate exocrine pancreatic insufficiency	100–200 µg/g
Severe pancreatic insufficiency	<100 µg/g

**Table 2:** Summary of patient-related measured vitamin D values in the group of patients with normal exocrine pancreatic function (A).

Range	Number (n = 88)	Percent (%)
Potentially harmful	3	3
Target range	26	30
Insufficiency	15	17
Deficiency	16	18
Severe deficiency	28	32

**Table 3:** Patient distribution by vitamin D levels in patients with elastase 1 levels below 200 µg/g (B).

Range	Number (n = 15)	Percent (%)
Potentially harmful	0	0
Target range	1	6
Insufficiency	8	53
Deficiency	2	13
Severe deficiency	4	27

**Table 4:** Baseline characteristics and vitamin D increase during short-term substitution in patients with and without exocrine pancreatic insufficiency.

Characteristic	EPI: Yes (10)	EPI: No (38)	*p*-Value
Demographic data			
Age (Years), mean (SD)	84.35 ± 6.15	82.6 ± 5.51	0.21
Gender, n (%)			
Male	4 (40%)	16 (42%)	
Female	6 (60%)	22 (58%)	
Clinical and laboratory parameters			
Vitamin D (µg/mL), mean (SD)	20.7 ± 11.35	23.4 ± 17.1	0.491
Vitamin D increase on day 12 (µg/L), mean (SD)	3.6 ± 2.32	7.2 ± 4.7	0.024
Kidney function as eGFR (mL/min/1.73 m^2^), mean (SD)	57.1± 19.1	56.9 ± 15.9	0.971
Total calcium (mmol/L), mean (SD)	2.17 ± 0.08	2.18 ± 0.08	0.799
Parathormone (ng/L)	65.7 ± 35.7	69.4 ± 33.8	0.777
Serum albumin (g/dL), mean (SD)	30 ± 2.22	33 ± 2.8	0.003
Calcium corrected for albumin (mmol/L), mean (SD)	2.31 ± 0.11	2.43 ± 0.11	0.012

**Table 5:** Renal function parameters classified in eGFR categories.

eGFR-Category (KDIGO 2024)	Range (mL/min)	Number of Patients	Share (%)
G1 (normal)	≥90	17	12%
G2 (slightly decreased)	60–89	62	42.5%
G3 (moderately-severely decreased)	30–59	50	34.5%
G4 (severely decreased)	15–29	16	11%

## Data Availability

The raw data supporting the conclusions of this article will be made available by the authors upon request.

## Abbreviations

The following abbreviations are used in this manuscript:

EPI	Exocrine pancreatic insufficiency
DRKS	Deutscher Register Klinischer Studien/German Clinical Trials Register
CKD	Chronic kidney disease
CP	Chronic pancreatitis
DVO	Dachverband Osteologie/German umbrella organisation for Osteology
GIT	Gastrointestinal tract
KDIGO	Kidney Disease: Improving Global Outcomes
CKD-EPI	Chronic Kidney Disease Epidemiology
CT	Computed tomography
MRT	Magnetic resonance tomography
UVB	Ultraviolet B
